# Functional imaging of the human brain using a modular, fibre-less, high-density diffuse optical tomography system

**DOI:** 10.1364/BOE.7.004275

**Published:** 2016-09-27

**Authors:** Danial Chitnis, Robert J. Cooper, Laura Dempsey, Samuel Powell, Simone Quaggia, David Highton, Clare Elwell, Jeremy C. Hebden, Nicholas L. Everdell

**Affiliations:** Biomedical Optics Research Laboratory, Department of Medical Physics and Biomedical Engineering, University College London, London, WC1E 6BT, UK

**Keywords:** (170.6960) Tomography, (170.0110) Imaging systems, (230.0250) Optoelectronics

## Abstract

We present the first three-dimensional, functional images of the human brain to be obtained using a fibre-less, high-density diffuse optical tomography system. Our technology consists of independent, miniaturized, silicone-encapsulated DOT modules that can be placed directly on the scalp. Four of these modules were arranged to provide up to 128, dual-wavelength measurement channels over a scalp area of approximately 60 × 65 mm^2^. Using a series of motor-cortex stimulation experiments, we demonstrate that this system can obtain high-quality, continuous-wave measurements at source-detector separations ranging from 14 to 55 mm in adults, in the presence of hair. We identify robust haemodynamic response functions in 5 out of 5 subjects, and present diffuse optical tomography images that depict functional haemodynamic responses that are well-localized in all three dimensions at both the individual and group levels. This prototype modular system paves the way for a new generation of wearable, wireless, high-density optical neuroimaging technologies.

## 1. Introduction

Diffuse optical tomography (DOT) is an extension of near-infrared spectroscopy (NIRS) [1] in which multiple sources and detectors of near-infrared light are arranged so as to provide a wide range of source-detector separations and overlapping spatial sampling of the target object [2]. The resulting multi-channel data can be used to reconstruct three-dimensional images of changes in the optical properties of that target object. When applied to the human scalp, DOT can be used to produce three-dimensional maps of changes in the concentration of the principal absorbers of near-infrared light in the human tissues, namely the oxygenated and de-oxygenated forms of haemoglobin [3]. It is this capacity to non-invasively image cerebral haemodynamics (and to do so using relatively inexpensive, portable equipment) that makes continuous-wave DOT a highly effective technology for functional imaging of the human brain [4,5].

While DOT has numerous advantages, its uptake as a functional neuroimaging methodology has been limited by several fundamental challenges. These include the technique’s high sensitivity to haemodynamics in the superficial, extra-cerebral tissues [6] and the necessity of obtaining appropriate anatomical spatial priors for optical image reconstruction. To address these challenges, superficial-signal regression techniques (which make use of the shallow sensitivity profile of channels with short source-detector distances (< 15 mm)), are becoming increasingly prevalent [7,8]. Meanwhile, the use of registered, subject-specific MRI images or (where no subject-specific MRI is available) generic atlas models as spatial priors for optical image reconstruction have been extensively tested and shown to be highly effective [9]– [11]. However, the greatest limiting factor in the acceptance of DOT as a functional neuroimaging method has been the need to balance the competing factors of sampling density, resolution, field-of-view and the practical challenge of applying high numbers of optical fibre bundles to the scalp of a subject [12].

In 2007, Zeff et al. [13] were the first to demonstrate what has become known as ‘high-density’ DOT. In their arrangement, 24 source and 28 detector fibres were packed together with a nearest-neighbour source-detector separation of 13 mm, and a total of ~340 channels were achievable over a scalp area of ~54 × 125 mm^2^. This was sufficient to cover the majority of the primary visual cortex. This significant improvement in sampling density allowed meaningful separation of the superficial and cortical layers in the resulting DOT images, and also resulted in a noticeably improved lateral image resolution [4]. In 2012, Habaermehl et al. used 30 optical fibres arranged over a small area of the parietal lobe to demonstrate that high-density DOT (which in this case incorporated so-called ‘null distance’ channels) can distinguish the functional response to somatosensory stimulation of two different digits on the same hand [14]. In 2014, Eggebrecht et al., extended the Zeff system to incorporate 188 optical fibre bundles, which allowed a greater proportion of the cortex to be sampled while maintaining the same high sampling density [15]. In doing so, they were able to demonstrate that DOT can be used to accurately map higher-level, distributed brain function and extended functional networks at the individual and group levels.

It is therefore evident that increasing sampling density yields a significant benefit to DOT; it facilitates the separation of scalp and brain signals and improves image resolution. However, the major advantages of DOT have always been that it is portable, can be easily applied in a range of environments and is well tolerated by infants, children and vulnerable patients. Despite their benefits, the current generation of high-density DOT systems risk undermining these advantages because of their increasingly bulky optical fibre arrangements. It is therefore apparent that achieving a light-weight, fibre-less, high-density DOT system (that can ultimately become a wearable device) is a critical next step in the development and acceptance of optical neuroimaging methodologies.

The miniaturization of NIRS technologies, and the development of fibre-less systems has long been pursued [16]. Early approaches used optical sources (typically LEDs) and detectors (typically photodiodes) coupled directly to the scalp, with the associated analogue signals being transferred via electrical cabling to a controller module [17–19]. This approach is clearly limited, as not only is the cabling itself cumbersome, the transfer of analogue data in this manner often leads to issues with RF noise. This issue has been overcome in several cases by placing the acquisition and analogue-to-digital conversion adjacent to the optical components at the scalp. This approach has been moderately successful, and there are now several fibre-less NIRS systems commercially available, including some that have been made completely wireless.

However, the majority of these systems exhibit a low dynamic range, and as such can provide only a single, fixed source-detector separation [20]. A limited dynamic range may also explain why almost all of these technologies are designed to measure only the pre-frontal cortex, where hair is not a confounding factor. By contrast, the NIRSport system developed by NIRx Medical Technologies LLC (NY, USA) can provide 16 sources and 16 detectors that can be placed anywhere over the scalp, but the system exists in a backpack-mounted format and cannot be used to obtain high-density measurements [5]. These technologies are therefore far from ideal if the goal is to produce functional *images* of the brain; they provide low sampling density and a narrow range of source-detector distances, which limits their suitability for tomographic image reconstruction. Furthermore, the majority of these technologies cannot be readily scaled up to sample multiple cortical areas.

Early in 2016, Choi et al. described the development of an integrated circuit for multi-channel NIRS, which was used as the basis for a 42-channel NIRS system that is also designed to sample only the pre-frontal cortex [21]. While this system is extremely promising, it is unclear from this sole publication whether it provides measurements at multiple source-detector distances, how its sampling density compares to fibre-based systems or whether the system can be used to produce 3D, tomographic images.

In this paper we present the first functional DOT images of the human brain obtained using a fibre-less, high-density DOT system, which were preliminarily disclosed at OSA BIOMED 2016 [22]. We describe the basis of our technology and demonstrate its efficacy in the measurement and imaging of the functional haemodynamic response in the adult human motor cortex.

## 2. Methods

### 2.1 The micro-NTS: a modular fibreless high-density DOT system

**Fig. 1:**
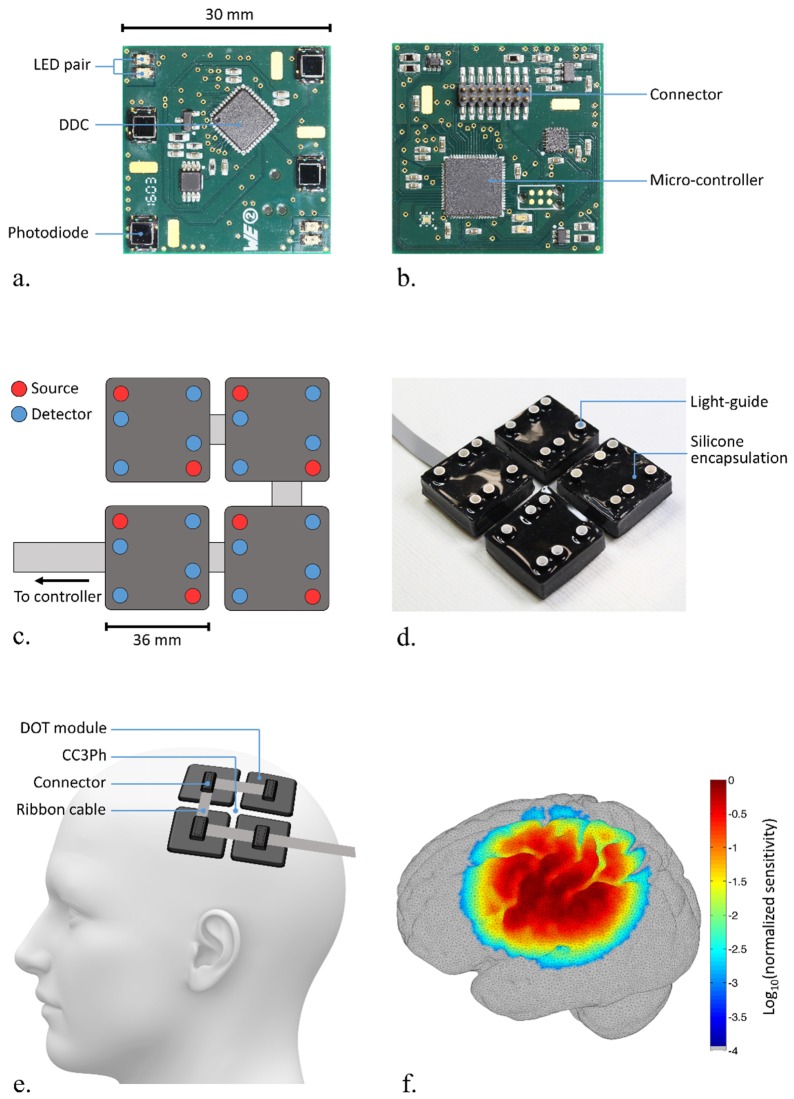
The uNTS modular DOT system. The module PCB is shown in front (a) and rear (b) views. Panel (c) depicts the array layout created with 4 uNTS modules, with a comparable photograph showing the encapsulated system in (d). Panel (e) shows the approximate arrangement of the 4-module uNTS on the scalp. The normalized array sensitivity, as calculated at 770 nm in a registered head mesh (Subject 4) for all theoretically available channels is shown in panel (f).

The system employed in this study (known as the micro-NTS (μNTS)) consists of multiple independent DOT modules, each constructed from a 30 × 30 mm PCB, 4 photodiodes (Hamamatsu, Japan) and 2 dual-wavelength sources comprised of 770 and 855 nm LEDs (OSA-Opto, Germany) (Fig. 1(a) and 1(b)

). Each photodiode is connected to the analogue input of a charge-to-digital converter (DDC114, Texas Instruments, USA), which includes a charge integration amplifier and a 20-bit Delta-Sigma analogue-to-digital converter (see Chitnis et al. [23], for more information).

The electronics are protected and made suitable for direct application to the scalp using a bespoke, multi-layered encapsulation (Fig. 1(c) and 1(d)). A 1 mm neoprene rubber sheet is applied over the PCB to optically isolate the LEDs and photodiodes so as to prevent internal light-piping from source to detector. The PCB and neoprene layer are encased in acrylic, which in turn is encapsulated within a black-pigmented silicone rubber. To couple light to and from the scalp, purpose-made light guides with a diameter of 3.5 mm were embedded in the encapsulation so as to protrude from the surface by 4 mm (Fig. 1(d)). Including this protrusion, each module has a cross-sectional height of 14 mm. Each module weighs approximately 15 g.

Four μNTS modules were used in this study, providing a total of 8 dual-wavelength sources and 16 detectors. The modules can be arbitrarily arranged to best suit the experimental requirements. Here, the 4 modules were arranged in a 2 × 2 grid pattern so as to provide up to 128 dual-wavelength channels, with a source-detector distance range of 8.5 to 85 mm (Fig. 1).

The system operates in a time-encoded arrangement with a default 20 ms integration time for each LED illumination. Acquisition control and timing information is sent, and digitized data are received, via a single ribbon cable that connects all μNTS modules to a controller, which at present is situated on-bench. In this study, the controller acquired a full frame of data (8 × 2 sources, plus a dark measurement) in 0.34 s, equivalent to a sample rate of 2.94 Hz. The acquired data is then sent to a PC via a USB-UART interface.

### 2.2 Functional imaging paradigm

As a first in-vivo test of this modular, fibre-less, high-density DOT system, 6 adult volunteers (5 male) with an age range of 23-51 years were recruited to take part in a functional imaging study, which was approved by the UCL Research Ethics Committee.

Each subject was seated, and after measurements of the head were obtained, the μNTS system was placed carefully over the primary somatomotor cortices contralateral to the subject’s dominant hand. The centre point of the 4 modules was positioned at CC3Ph (or CC4Ph for theone left-handed participant) in the international 10-5 coordinate system (Fig. 1(e)), with the vertical edges of the modules aligned parallel to the line between Cz and the left (or right) pre-auricular point. Once positioned, the array was coupled to the head using a simple bandage, which was then covered with a dark cloth. Note that beyond briefly moving the modules from side-to-side, no effort was made to remove hair from underneath the optical contacts.

To facilitate accurate spatial registration, each individual’s cranial landmarks, and the position of the corners of each μNTS module were measured using a Polhemus Patriot 3D digitizer (VT, USA) and an in-house GUI.

The stimulation protocol consisted of a classic motor cortex stimulation experiment. Two runs of 20, 15-second blocks of a dominant-hand, thumb-to-finger extension task were interspersed with periods of rest of a pseudo-random duration ranging from 15 to 20 seconds. The paradigm timing was controlled by a Matlab script (Mathworks, USA) that provided visual cues on the screen of a laptop positioned in front of the subject. This script also allowed the DOT data acquisition to be time-locked to the stimulus paradigm. During the experiment, the subject was instructed to remain as still as possible and the lighting level in the room was reduced. Note that one subject (a male) was removed from further analysis because of a noticeable shift in the array during data collection.

### 2.3 DOT data pre-processing

Intensity data, measured in parts-per-million of full scale (PPM-FS), were recorded from each photodiode for illumination of each of the 16 LEDs plus a dark measurement, corresponding to a total of 272 measurements per frame. The dark measurement obtained from each photodiode was subtracted from all other measurements for that photodiode on a frame-by-frame basis. To assess the measured signal as a function of source-detector distance, both the raw and dark-corrected intensity measurements (in PPM-FS) were converted to optical power (in Watts) by calculating the equivalent current associated with each measurement and multiplying by the spectral responsivity of the photodiode.

A total of 128 channels of dark-corrected, dual-wavelength data were restructured into the Homer2 [24] data format for initial examination and processing. To obtain haemodynamic response functions (HRFs) in each subject, the dark-corrected data was converted to optical density, then low-pass filtered at 0.5 Hz. Periods of motion artifact were marked by visual inspection, and any stimulus trial concurrent with those periods was rejected. The ratio of the standard deviation of an intensity measurement to its mean (SDMratio) was also used as a measure of signal quality. Two different thresholds of this ratio were used to exclude data. In the first case, channels were rejected if the SDMratio exceeded 7.5%, which is a value that has been used repeatedly in previous studies [25]. The second (less conservative) processing stream rejected channels if the SDMratio exceeded 25%. Channel-wise HRFs were obtained for each subject by converting changes in optical density to changes in oxy- and deoxy-haemoglobin concentration (from herein HbO and HbR respectively) using the modified Beer-Lambert law prior to a simple block-average over stimulus trials and runs. The HRF epoch window was set as from −5 to 25 s relative to stimulus onset.

### 2.4 Head modelling and image reconstruction

The digitized anatomical landmark positions (namely the inion, nasion, left and right pre-auricular points and the vertex) recorded in each subject were used to register a mesh derived from the MNI 152 adult MRI atlas [26] to each subject’s specific coordinate space. This mesh contains of 218,000 nodes, with a median Voronoi volume of less than 15 mm^3^. This mesh package is freely available at www.ucl.ac.uk/medphys/research/adultMNImodel. The registration process yielded a spatially-registered, 5-layer (scalp, skull, CSF, grey matter, white matter), finite-element mesh for each subject. Using the digitized corner points of the μNTS modules, the location of each optical source and detector position could be derived for each subject.

Volumetric images of HbO and HbR were reconstructed in two stages. To begin, the change in the absorption coefficients at each wavelength, and at each point in time, were found by a minimization of the form:

$$\Delta \mu_{a}^{\delta }={\text{arg min}}_{\mu_{a}}E(\mu_{a})≔{\left\|y^{\delta }-J\mu_{a}\right\|}_{\Gamma_{e}^{-1}}^{2}+\lambda R(\mu_{a}),$$ (1.1)

where *y^δ^ = y*^obs^ *– y*^ref^, *y*^obs^ is the measured data, *y*^ref^ is a reference measurement taken under assumed parameters *μ_a,0_*, *J = f’*(*μ_a,0_*) is the Fréchet derivative of a suitable forward model of light propagation evaluated at the reference parameters, Γ*_e_* is the covariance matrix of the data, and *λR*(*μ_a_*) is a scaled regularization function which serves to overcome the ill-posedness of the problem by the introduction of prior knowledge.

In this case, the measured data was the natural logarithm of the change in intensity at each wavelength, averaged over accepted trials. The covariance matrix Γ_e_ = I was chosen according to the assumption of independent Gaussian noise of constant relative magnitude [27]. The reference measurement *y*^ref^ was taken as the mean value of the first five seconds of the measured data (i.e. the period prior to stimulus onset). We employed first order Tikhonov regularization such that *R*(*μ_a_*) *= ||*∇*μ_a_||^2^*, consistent with the assumption of smooth changes in the distribution of *μ_a_* throughout the measurement volume. The hyper-parameter *λ* was chosen by inspection to be 0.01 and fixed across all time points and subjects. This value is consistent with the range typically employed in DOT studies [15]. A finite element discretisation of the diffusion approximation was used as a forward model, constructed using the TOAST + + software package [28]. Minimization of E(*μ_a_*) was performed using the conjugate gradient method [29].

Images of changes in *μ_a_* at the two wavelengths were converted into images of HbO and HbR using the specific absorption coefficients provided by the Homer2 processing package [24]. The reconstruction process yielded an 84-frame image sequence for both HbO and HbR in FEM space for each subject. For visualization purposes, these volumetric mesh images were projected to a registered grey-matter surface mesh by taking a mean of the volumetric image values contained within a 3 mm radius spherical kernel centred at the location of each grey-matter mesh node. As each individual’s anatomical space was a registered version of the MNI152 adult atlas, group-level analyses were made possible in both the volume and grey-matter surface meshes by simply transferring each individual’s image sequence to the original atlas mesh on a node-to-node basis. Note that in the case of the single left-handed subject, all forwarded modelling and reconstruction was performed in the right hemisphere of that subject’s registered mesh, but for visualization and group-level analysis, the resulting images were transferred to the left hemisphere by exploiting the symmetry of the selected MNI atlas model. To prevent any one response biasing the group-level images, each subject’s HbO and HbR images were normalized to their own maxima prior to averaging. Note that all displayed images are thresholded at 60% of their maximum value.

## 3. Results

### 3.1 Pre-processing and measures of DOT data quality

Figure 2

**Fig. 2:**
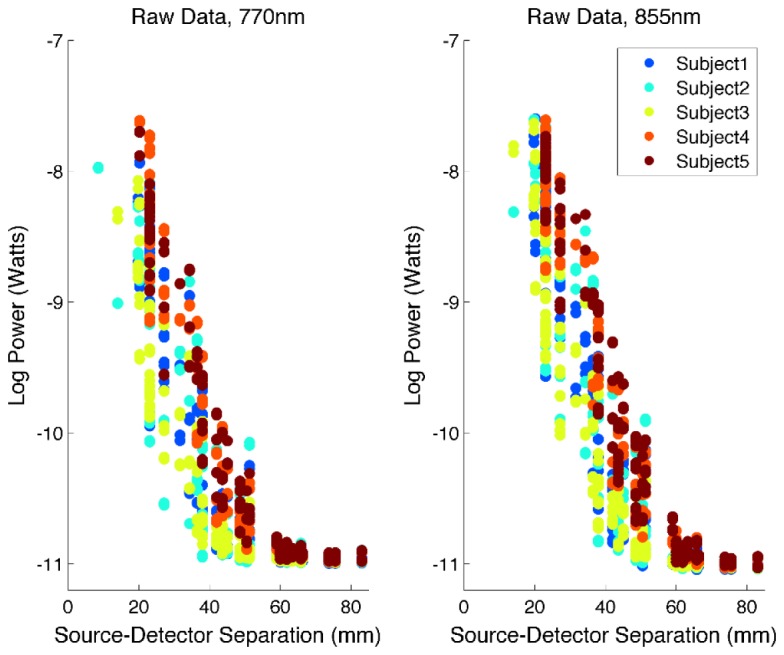
The raw log (base 10) measured power, averaged over time-points, plotted as a function of source-detector separation in all 5 subjects for 770 nm sources (left) and 855 nm sources (right).

shows the raw measured optical power as a function of source-detector separation for all 5 subjects. Figure 3

**Fig. 3:**
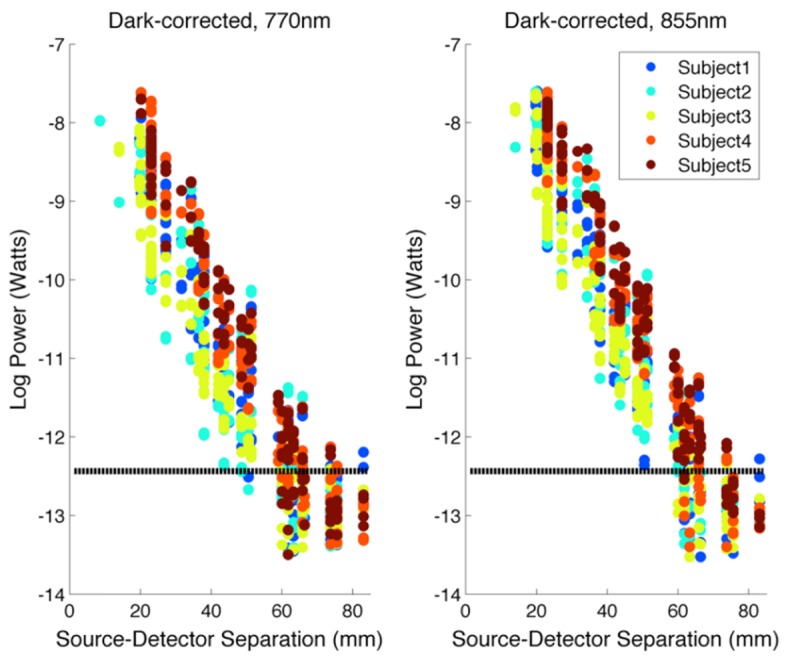
The log (base 10) dark-count corrected power, averaged over timepoints and plotted as a function of source-detector separation for all 5 subjects for 770 nm sources (left) and 855 nm sources (right). The estimated noise equivalent power of 370 fW is also depicted as the black dashed line. Note that points below this level are apparent because an average has been taken over time. None of these measurements are used in further analysis.

shows the dark-corrected power measurements as a function of source-detector separation. The theoretical minimum detectable power of this system (i.e. that equivalent to 1 PPM-FS) is 31 fW at 770 nm. However, the median standard deviation of the dark measurements provides an empirical and realistic estimate of the noise-equivalent power of our system. Across all 5 subjects, the median standard deviation of all dark measurements value was found to be 370 fW. The maximum detectable power (i.e. 1e6 PPM-FS) corresponds to 31nW. The μNTS system therefore provides a true dynamic range of 98.6 dB, as calculated using real experimental data recorded in adults. Note that this dynamic range marks a significant improvement on the previous design [23]. This was primarily achieved throughimproved timing of the integration and clock signals, which reduces the influence of both fluctuations in the device's offset and noise due to the 50 Hz (UK) mains power supply.

**Fig. 4:**
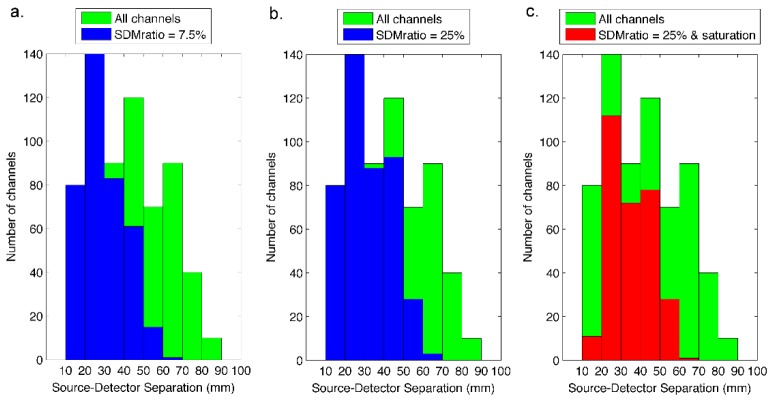
Histograms of all possible channels and accepted channels across all 5 subjects as a function of source-detector separation. Panel a) compares the distribution of all possible channels (green) with that of channels that passed the signal-to-noise pruning process with and SDMratio threshold of 7.5% (blue). Panel b) displays the same but with an SDMratio threshold of 25%. Panel c) displays the same as b), but with additional channels removed due to saturation effects.

From a theoretical maximum of 128 channels, an average of 75 channels (range 65-87) survived the data pruning process at a SDMratio threshold of 7.5% (Fig. 4(a)

). An average of 86 channels survived when this threshold was increased to 25% (Fig. 4(b)). This less-conservative approach was applied because lower thresholds were found to remove channels that (after processing) yielded good-quality HRFs. This is likely explained by the relatively high number of stimuli repetitions performed in this study. Note that 34 of the 128 channels in the array had a source-detector separation of greater than 60 mm, meaning that on average, 86/94 (91.4%) of channels with a source-detector separation less than 60 mm were sufficiently low-noise to survive the data-pruning process.

In addition to channel rejection on the basis of noise, a number of channels in each subject had to be rejected due to optical saturation of a detector. This occurred primarily in the shortest (8.5 mm) separation channels. It was also necessary in some cases to remove channels where the detector had been saturated in the previous illumination cycle. The total number of channels taken forward for further analysis therefore ranged from 59 to 65, with a median of 61 (Fig. 4(c)). The total number of stimulation trials rejected due to visually-identified motion artifact was 42 from a total of 200. The minimum number of trials included in any one subject was 22 out of a possible 40 (Subject 4).

Figure 5

**Fig. 5:**
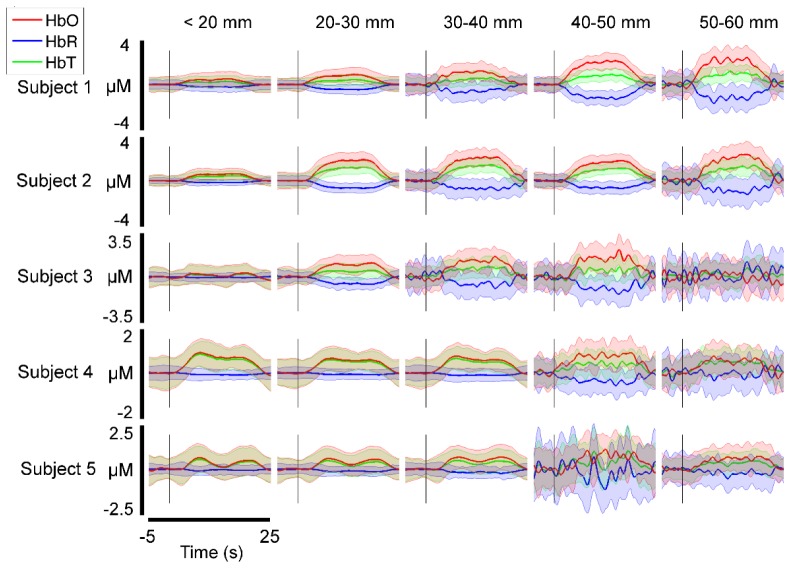
Selected HRFs for each subject displayed as a function of source-detector separation. The lighter-coloured, shaded areas depict standard error across trials. These channels were selected on the basis that they show the largest increase in HbO of any channel in each source-detector separation range.

shows example HRFs for all five subjects as a function of source-detector separation. Note that clear haemodynamic responses are apparent in all subjects, and in 4/5 cases, responses are apparent at a separation greater than 50 mm. In all but one subject (Subject 4), the largest amplitude HbO response is apparent at a separation above 20 mm, with a smaller response visible in channels below 20 mm, which have a higher relative sensitivity to superficial tissues [30].

Figure 6

**Fig. 6:**
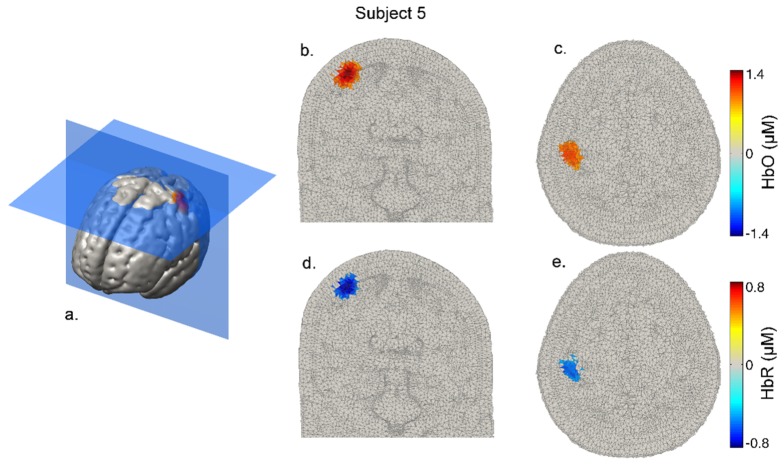
Volumetric HbO and HbR images at 12 seconds post-stimulus-onset in subject 5. Panel a. provides an indication of the slice locations, while b. and c. show the HbO images in coronal and transverse section and d. and e. show the same for HbR. Note that the response is well localized in the superficial cortex.

shows coronal and transverse views of the reconstructed DOT images of HbO and HbR at 12 seconds post-stimulus-onset in a representative subject (subject 5). In this subject, 57 channels (8 with a separation > 50 mm) survived to contribute to image reconstruction. Figure 7

**Fig. 7:**
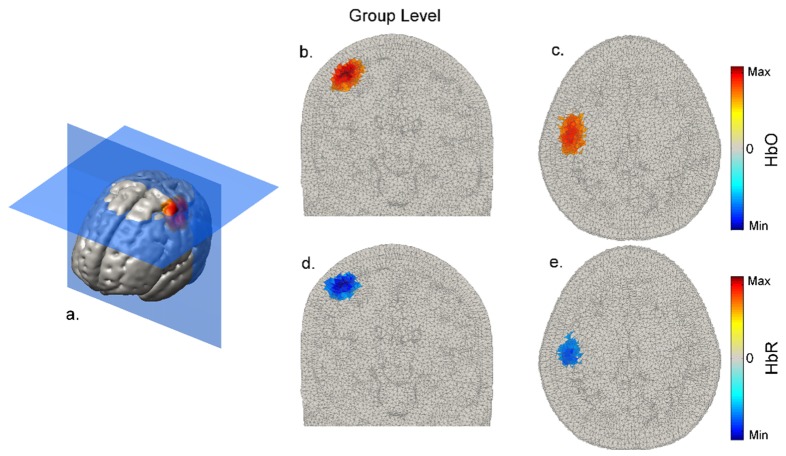
Normalized and group-averaged, volumetric response images at 12 seconds post-stimulus-onset. Panel a. provides an indication of the slice locations, while b. and c. show the HbO images in coronal and transverse section and d. and e. show the same for HbR. Note that the response is localized in all three dimensions.

depicts the equivalent at the group level. Note the clear, co-incident increases in HbO and decreases in HbR, which are localized in all three dimensions. Figure 8

**Fig. 8:**
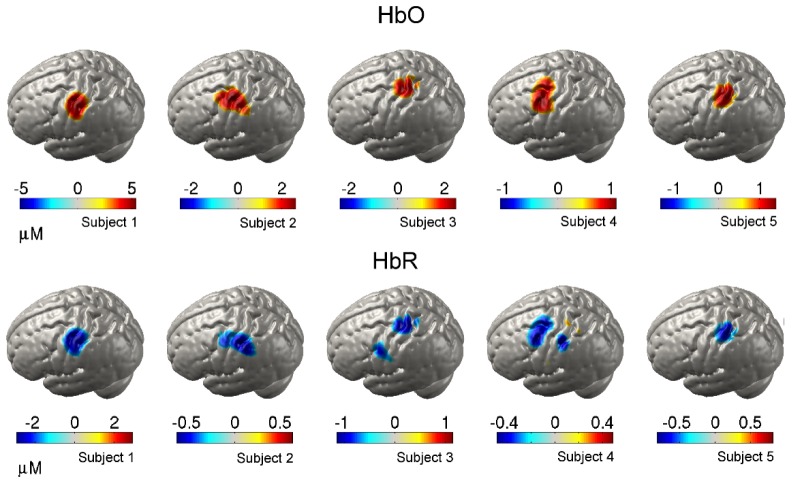
Grey-matter surface images of HbO and HbR at 12 seconds post-stimulus-onset for all 5 subjects.

. shows the cortical projections of the HbO and HbR DOT images at 12 seconds post-stimulus-onset of all 5 subjects. While there is some variation, all demonstrate increases in HbO and (smaller amplitude) decreases in HbR that occupy only a small volume of the system’s field of view (see Fig. 1(b)). In all but Subject 4, both the peak HbO and peak HbR changes occur within the pre- or post-central gyri.

Figure 9

**Fig. 9:**
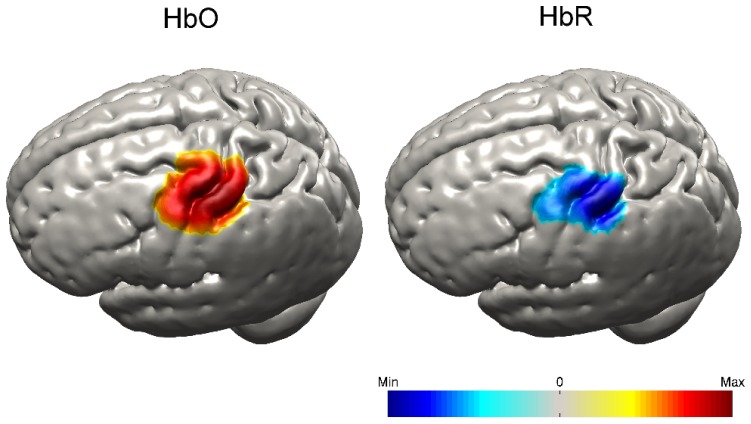
Normalized and group-averaged grey-matter surface images of HbO and HbR at 12 seconds post-stimulus-onset.

shows the group-average cortical projection images of HbO and HbR at 12 seconds post-stimulus-onset. At the group level, a localized response is also apparent, with the peak HbO and peak HbR changes both occurring across the pre and post-central gyri, at a cortical location that lies directly beneath 10-20 position C3. This location is consistent with the digit centres of the primary motor and somatosensory cortices, as identified by fMRI [31,32].

## 4. Discussion

Portability has always been a key advantage of NIRS and diffuse optical imaging techniques, and it has long been a goal of NIRS hardware research to develop effective wearable and wireless systems. The recent and rapid development of DOT systems that provide a high sampling density, and the significant advantages they exhibit, has further driven the need for diffuse optical imaging electronics to be sufficiently minimized to allow high-density measurements in a truly wearable form-factor.

While numerous fibreless NIRS and multi-channel NIRS systems have been demonstrated and commercialized [5], to our knowledge this is the first time a high-density, fibreless DOT system has been demonstrated *in-vivo*. The μNTS prototype system is extremely light-weight, and the encapsulation ensures it is comfortable and well-tolerated. The system’s novel modularity is also highly advantageous for functional imaging research: the design allows modules to be arranged in an arbitrary fashion so as to suit the demands of any experiment, and the system can theoretically permit up to 75 modules to be used simultaneously, with only a single cable connecting the modules to one-another and the controller. This is more than sufficient to cover the entire adult scalp.

Another key novelty of this technology is its outstanding sensitivity and dynamic range. A large dynamic range is important for NIRS systems, but is critical for DOT approaches, where a wide range of source-detector separations is needed to enable the reconstruction of three-dimensional images of the brain and the separation of haemodynamic effects in the superficial tissues. This system demonstrates a measured (rather than theoretical) dynamic range of 98 dB. Measurements above the noise-equivalent power are achieved at source-detector separations up to and above 70 mm (Fig. 3), and data that was sufficiently high-quality to contribute to the image reconstruction process was acquired at separations > 55 mm in all subjects, and at separations > 60 mm in one subject. This is despite the presence of hair, and with little effort being made to move hair from underneath the optical contacts. The data quality obtained by the μNTS at these longer separations therefore appears superior to that of comparable fibre-based high density systems [9,15].

The ability to obtain data from such a wide range of source-detector separations does, as one would expect, result in high quality DOT images, as illustrated by Figs. 6–9. The increase in HRF amplitude as a function of source-detector separation that is evident in Fig. 5 translates to functional haemodynamic response images that are localized in depth, as well as in the plane of the μNTS array. This was achieved without pre-reconstruction regression of short-separation channels using a reconstruction process that makes no specific assumptions about the form of the superficial response. Furthermore, depth-resolved images of cerebral activation were achieved despite the fact that the majority of channels with a separation of less than 15 mm had to be excluded due to saturation of the detectors.

This issue of saturation of channels with a separation of < 15 mm is a significant limitation because the inclusion of these channels is likely to further improve the differentiation between scalp and cerebral haemodynamics [4,30]. In future iterations of this technology, this problem can be readily solved (without compromising the system’s high sensitivity) by altering the timing control design of the LEDs and by taking advantage of variable integration times. This approach should make it possible to increase the system’s effective dynamic range by at least 2 orders of magnitude.

We have also already begun to expand this technology, with the goal of producing a wearable high-density system that provides full scalp coverage. This will require significant developments in control and synchronization; because the detector methodology precludes the use of frequency modulation, future systems must necessarily use a combination of temporal and spatial multiplexing. Achieving the most efficient combination of these two approaches can be modelled as a numerical optimization problem. The illumination protocol could therefore be adapted for each specific array configuration and application, and it may even be possible to develop dynamic approaches that seek to continuously optimize data quality during acquisition. We are also already developing multi-wavelength versions of this technology that allow changes in the concentration of additional tissue chromophores (including cytochrome-c-oxidase) to be examined [23]. Using a suitable communication protocol and standard wifi or Bluetooth approaches, this technology can also be readily adapted to become fully wireless.

## 5. Conclusion

The ability to obtain high-quality, 3D images of human brain function, with a technology that is both wearable and wireless and has a wide field-of-view will have a significant impact on clinical and cognitive neuroscience. The high sensitivity, large dynamic range and outstanding in-vivo data quality provided by the prototype μNTS system demonstrates its great potential as a functional neuroimaging tool.
